# Intrasystem Repeatability of S-Detect for Breast Ultrasound Classification With Identical Static Images: Single-Center Retrospective Repeatability Study

**DOI:** 10.2196/86278

**Published:** 2026-07-03

**Authors:** Liang Yongping, Ping Zhou, Yang Wang, Nan Zhang, Qing Zhou, Xinghao Zhang, Haifeng Cai, Zhang Juan

**Affiliations:** 1 Department of Ultrasound, Tangshan People's Hospital, Tangshan, China; 2 Department of Ultrasound, Third Xiangya Hospital, Changsha, China; 3 Department of Breast Surgery, Tangshan People's Hospital, Shengli Road #65, Tangshan, 063000, China, 86 13393159122

**Keywords:** breast cancer, ultrasonography, computer‑aided diagnosis, repeatability, S‑Detect

## Abstract

**Background:**

Computer-aided diagnostic systems such as S-Detect (Samsung Medison) are increasingly integrated into breast ultrasound workflows. Notwithstanding extensive past evaluation of S-Detect’s diagnostic accuracy, its intrasystem repeatability at the software level with identical static images, a fundamental prerequisite for clinical reliability, has not been systematically investigated.

**Objective:**

This study aimed to evaluate the intrasystem repeatability of the S-Detect computer-aided diagnostic system in classifying breast nodules in identical static ultrasound images.

**Methods:**

This retrospective, registered, blinded repeatability study analyzed 398 breast nodules from 261 women (mean age 43.10, SD 12.57 years) who underwent surgery between February 2019 and March 2020 at a single institution. Identical stored static ultrasound images, acquired by a single experienced sonographer on 1 Samsung RS80A ultrasound system, were each analyzed twice using the same S-Detect workstation: immediately after acquisition (S-Detect 1) and again at least 4 weeks later under blinded conditions with manual cursor repositioning (S-Detect 2). Repeatability was assessed using concordance rate and Cohen κ. The diagnostic performance of each run was compared against surgical histopathology.

**Results:**

Of 398 nodules, 156 (39.2%) were initially classified as possibly benign, and 242 (60.8%) were initially classified as possibly malignant. On repeat analysis, 37.4% (149/398) and 62.6% (249/398) of the nodules were classified as possibly benign and malignant, respectively. A total of 4.5% (7/156) of the nodules initially classified as benign were reclassified as malignant, whereas no malignant-to-benign changes occurred. The overall concordance rate was 98.2% (391/398), with a Cohen κ of 0.95 (95% CI 0.94‐0.99; *P*<.001). Diagnostic performance remained stable across runs (area under the curve=0.913 vs 0.902; *P*=0.702).

**Conclusions:**

Under controlled conditions with identical static images, S-Detect showed high intrasystem repeatability, underscoring strong software-level consistency, although its translation to real-world clinical reproducibility requires further validation.

## Introduction

Breast cancer is the most common malignancy among women and remains a leading cause of cancer-related morbidity and mortality worldwide [1]. In China, it ranks first among female cancers, but China differs from Western countries in epidemiology, with a younger age of onset, later stage at diagnosis, and distinct pathological subtypes [2,3]. These features complicate prevention and management, underscoring the importance of early detection for improving outcomes [4,5].

Mammography is the primary imaging tool for breast cancer screening, yet its sensitivity declines in dense breasts, a feature more common among Asian women. Ultrasound is widely used as an adjunct because it avoids ionizing radiation and has superior sensitivity for solid lesions [6-9]. However, interpretation of conventional breast ultrasound is operator dependent, subject to intra- and interobserver variability, and limited by image repeatability [10].

Computer-aided diagnosis (CAD) and artificial intelligence have shown promise in medical imaging, including breast ultrasound, particularly for assisting less experienced readers [11-13]. Most prior studies, however, have emphasized diagnostic accuracy and radiologist concordance, with little attention to repeatability of CAD outputs when analyzing identical images [14]. Therefore, this study aimed to evaluate the intrasystem repeatability of the S-Detect CAD system (Samsung Medison) when identical stored breast ultrasound images were reanalyzed under standardized blinded conditions while distinguishing software-level repeatability from real-world clinical reproducibility.

## Methods

### Ethical Considerations

This retrospective, registered, blinded repeatability study was approved by the ethics committee of the Third Xiangya Hospital, Central South University (approval R19004) and was registered with the Chinese Clinical Trial Registry (ChiCTR-1800019649). All participants provided written informed consent prior to enrollment.

### Participants

From February 2019 to March 2020, consecutive female patients scheduled for surgical excision of breast lesions at the Department of Breast and Thyroid Surgery, Third Xiangya Hospital, were screened for inclusion. Inclusion criteria were (1) female sex and age of 18 to 70 years, (2) presence of a breast nodule with definitive histopathology available from surgical specimens, (3) solid or predominantly solid lesion (>50% solid component), and (4) informed consent. Exclusion criteria included neoadjuvant therapy prior to surgery, prior core-needle biopsy or vacuum-assisted excision of the target lesion, presence of breast implants, inadequate sonographic visualization, or refusal to participate.

Of the initial 269 patients, 8 (3%) were excluded (3 discharged without surgery and 5 with prior minimally invasive surgery), leaving 261 (97%) patients and 398 nodules for analysis.

### Ultrasound Image Acquisition

All examinations were performed on a Samsung Medison RS80A Ultrasound system (Samsung Medison Co., Ltd) using a linear transducer (5-13 MHz; center frequency of 8.4 MHz). A single sonographer with 5 years of experience performed all scans. Imaging parameters (gain, depth, and focus) were standardized. For each lesion, the sonographer obtained orthogonal views and stored the largest cross-section as a static image, with the lesion occupying approximately one-quarter to one-fifth of the frame. Lesion characteristics (size, echotexture, margin, and location) were recorded.

### S-Detect Analysis

All stored ultrasound images underwent 2 independent analyses using the same workstation, ultrasound system, and software version. The first analysis (S-Detect 1) was performed immediately after image acquisition during the original ultrasound examination. After selecting the stored static image, the operator positioned the cursor approximately at the center of the lesion and activated the S-Detect function. Automatic lesion segmentation was then performed by the software. No manual adjustment of lesion boundaries was allowed. The system subsequently generated a binary classification result (“possibly benign” or “possibly malignant”), as shown in MultimediaAppendices 1^5^.

The second analysis (S-Detect 2) was performed at least 4 weeks after the initial assessment. The same stored image was reloaded and analyzed using the same workstation, ultrasound system, software version, and default settings. The operator was fully blinded to the initial S-Detect result and repeated the same procedure used in S-Detect 1, including cursor placement at the approximate lesion center followed by automatic segmentation. No manual boundary correction or additional image preprocessing was performed.

Therefore, the procedures used in S-Detect 1 and S-Detect 2 were identical. The only intended difference was the temporal separation and blinding of the second assessment. Any discrepancy between the 2 analyses, therefore, reflected intrinsic system-level variability rather than differences in image acquisition, software configuration, or operator interaction.

### Reference Standard

Histopathological diagnosis of the surgical specimen served as the reference standard for lesion classification (benign vs malignant). The primary study outcome was repeatability between S-Detect 1 and S-Detect 2. Secondary outcomes included diagnostic performance metrics of each S-Detect run against histopathology.

### Statistical Analysis

Statistical analyses were performed using SPSS (version 22.0; IBM Corp). Concordance between S-Detect 1 and S-Detect 2 was calculated as the percentage of lesions with identical binary classifications. Repeatability was quantified using the Cohen κ with 95% CIs and interpreted as poor (<0.20), fair (0.21-0.40), moderate (0.41-0.60), substantial (0.61-0.80), and almost perfect (0.81-1.00). Sensitivity, specificity, positive predictive value (PPV), negative predictive value (NPV), accuracy, and area under the curve (AUC) with 95% CIs were calculated for each S-Detect run vs histopathology. A *P* value of less than .05 was deemed statistically significant.

## Results

### Patient and Lesion Characteristics

**Figure 1.**
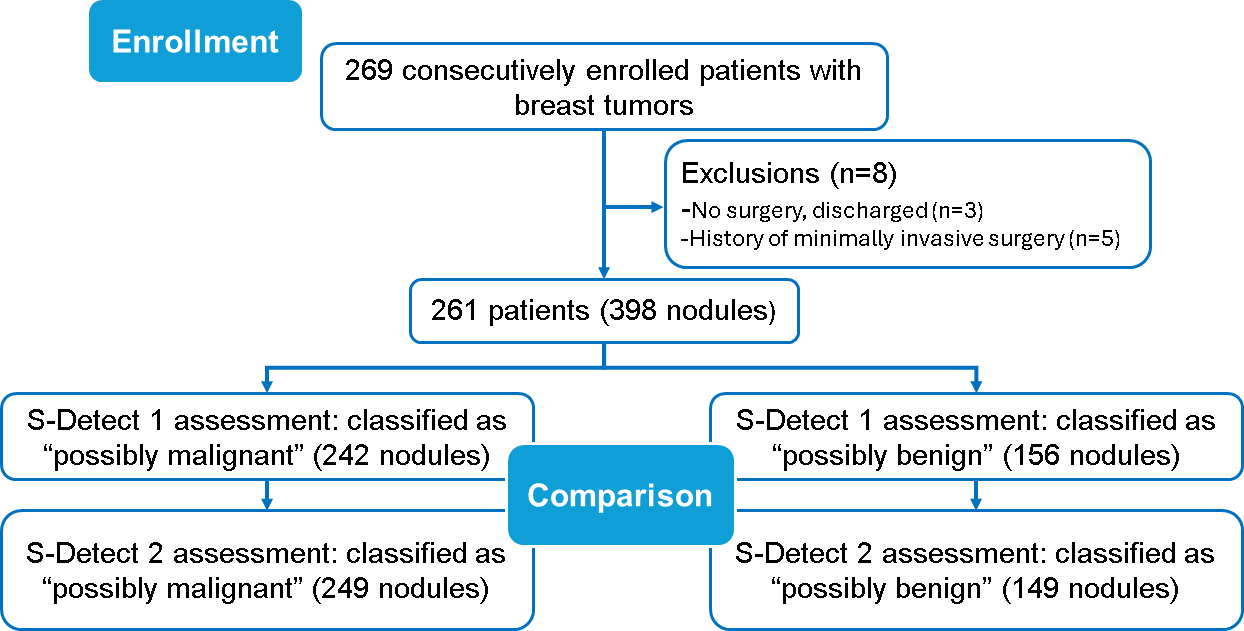
Workflow diagram. Arrows indicate the sequential steps from patient enrollment to outcome analysis.

This study included 261 female patients (mean age 43.10, SD 12.57; range 17-70 years) with 398 breast nodules (Figure 1). Mean maximum lesion diameter was 1.89 (SD 1.26; range 0.50-6.50) cm. According to the first S-Detect analysis, of the 398 nodules, 156 (39.2%) were labeled as “possibly benign” (mean diameter 1.33, SD 0.52 cm), and 242 (60.8%) were labeled as “possibly malignant” (mean diameter 2.18, SD 1.34 cm). Nodule distribution by laterality and quadrant is summarized in Table 1.

**Table 1.** Baseline characteristics of patients and breast nodules. “Possibly benign” and “possibly malignant” groupings are based on the first S-Detect analysis (S-Detect 1) only.

Item	Possibly benign nodules (n=156)	Possibly malignant nodules (n=242)	Total nodules (n=398)
Age (y)			
Mean (SD)	41.51 (10.76)	43.29 (12.84)	43.10 (12.57)
Median (IQR)	43.0 (13.0)	45 (16.0)	45 (15.0)
Maximum diameter of nodules (cm)			
Mean (SD)	1.33 (0.52)	2.18 (1.34)	1.89 (1.26)
Median (IQR)	1.10 (0.73)	2.02 (1.47)	1.60 (4.1.49)
Position of nodules, n (%)			
Left breast	79 (50.6)	133 (55.0)	212 (53.3)
Right breast	77 (49.4)	109 (45.0)	186 (46.7)
UOQ^a^	86 (55.1)	131 (54.1)	217 (54.5)
LOQ^b^	27 (17.3)	29 (12.0)	56 (14.1)
UIQ^c^	28 (17.9)	55 (22.7)	83 (20.9)
LIQ^d^	15 (9.6)	27 (11.2)	42 (10.6)

a UOQ: upper outer quadrant.

b LOQ: lower outer quadrant.

c UIQ: upper inner quadrant.

d LIQ: lower inner quadrant.

### Repeatability Between S-Detect 1 and S-Detect 2

On repeat analysis of the identical stored images, 4.5% (7/156) of the lesions originally classified as “possibly benign” in S-Detect 1 were reclassified as “possibly malignant” in S-Detect 2. No lesion initially classified as “possibly malignant” was reclassified as “possibly benign” on repeat assessment (Table 2). The overall concordance rate between the 2 runs was 98.2% (391/398) of the nodules. The Cohen κ demonstrated almost perfect repeatability (κ=0.95, 95% CI 0.94-0.99; *P*<.001).

**Table 2.** Concordance between the first (S-Detect 1) and second S-Detect (S-Detect 2) analyses. “Benign” and “malignant” indicate lesions classified as likely benign or likely malignant, respectively.

	S-Detect 2: benign, n (%)	S-Detect 2: malignant, n (%)
S-Detect 1: benign (n=156)	149 (95.5)	7 (4.5)
S-Detect 1: malignant (n=242)	0 (0)	242 (100)
Total (n=398)	149 (37.4)	249 (62.6)

### Comparison With Histopathology

Pathological examination identified 170 benign and 228 malignant nodules. In S-Detect 1, a total of 86.5% (147/170) of the pathologically benign lesions were correctly classified as benign, and 13.5% (23/170) were labeled as possibly malignant; 96.1% (219/228) of the malignant lesions were correctly classified as possibly malignant, and 3.9% (9/228) were labeled as possibly benign. In S-Detect 2, a total of 83.5% (142/170) of the benign lesions were labeled as benign, and 16.5% (28/170) were labeled possibly malignant; 96.9% (221/228) of the malignant lesions were labeled as possibly malignant, and 3.1% (7/228) were labeled as possibly benign (Table 3).

**Table 3.** Concordance between pathology and S-Detect analyses.

	Pathology: benign (n=170), n (%)	Pathology: malignant (n=228), n (%)
S-Detect 1^a^: benign	147 (86.5)	9 (3.9)
S-Detect 1: malignant	23 (13.5)	219 (96.1)
S-Detect 2^b^: benign	142 (83.5)	7 (3.1)
S-Detect 2: malignant	28 (16.5)	221 (96.9)

a S-Detect 1: initial assessment.

b S-Detect 2: repeat assessment.

Diagnostic performance metrics are shown in Table 4. S-Detect 1 achieved a sensitivity of 96.05% (95% CI 92.91%-98.02%), specificity of 86.47% (95% CI 80.29%-91.12%), PPV of 90.50% (95% CI 86.08%-93.78%), NPV of 94.23% (95% CI 89.10%-97.14%), accuracy of 91.96% (95% CI 88.91%-94.28%), and AUC of 0.913 (95% CI 0.887-0.939). S-Detect 2 produced comparable results (sensitivity=96.93%; specificity=83.53%; PPV=88.76%; NPV=95.30%; accuracy=91.21%; AUC=0.902); the difference in AUC was not statistically significant (*P*=0.702).

**Table 4.** Diagnostic performance of S-Detect in the first (S-Detect 1) and second (S-Detect 2) analyses against pathology.

Metric	S-Detect 1 (%; 95% CI)	S-Detect 2 (%; 95% CI)
Sensitivity	96.05 (92.91-98.02)	96.93 (93.93-98.58)
Specificity	86.47 (80.29-91.12)	83.53 (76.97-88.64)
PPV^a^	90.50 (86.08-93.78)	88.76 (84.13-92.21)
NPV^b^	94.23 (89.10-97.14)	95.30 (90.59-97.83)
Accuracy	91.96 (88.91-94.28)	91.21 (88.10-93.58)
AUC^c^	0.913 (0.887-0.939)	0.902 (0.875-0.929)

a PPV: positive predictive value.

b NPV: negative predictive value.

c AUC: area under the curve.

## Discussion

### Principal Findings

This study demonstrated almost perfect intrasystem repeatability of the S-Detect CAD system when identical static breast ultrasound images were reanalyzed under controlled conditions, with a concordance rate of 98.2% (391/398) and a Cohen κ of 0.95 across 2 blinded repeat analyses performed on the same device. Diagnostic performance also remained stable between the 2 analyses (AUC=0.913 vs 0.902; *P*=0.702), indicating strong software-level consistency. Importantly, these findings should be interpreted within the specific context of repeat analysis of identical stored images and should not be directly extrapolated to real-world clinical reproducibility during live ultrasound acquisition. Unlike software-level repeatability assessed under standardized conditions, clinical reproducibility is additionally influenced by multiple operator- and acquisition-related factors, including probe positioning, scanning technique, imaging parameter selection, lesion visualization, and patient-related variability. Accordingly, these findings primarily characterize software consistency under controlled conditions rather than procedural reproducibility during live clinical scanning.

Since the introduction of CAD technology more than half a century ago, sustained scientific and technological progress has markedly advanced its sophistication and facilitated its gradual integration into clinical practice, improving diagnostic objectivity and accuracy across modalities [15,16]. In breast ultrasonography specifically, CAD can provide reference information comparable to the judgments of experienced sonographers and can also serve as an educational resource for junior practitioners [17-19]. Most prior S-Detect research, however, has emphasized diagnostic accuracy and reader assistance effects compared with histopathology, particularly for less experienced radiologists, whereas the repeatability of CAD outputs on identical images, a prerequisite for clinical reliability, has been less systematically examined [20,21]. Our findings are consistent with the limited literature on CAD repeatability in mammography and extend those observations to ultrasound, a modality with inherently greater acquisition variability, by showing that, under standardized acquisition and a controlled analysis environment, a commercial integrated CAD module can be highly stable [22,23].

### Repeatability vs Clinical Reproducibility

These findings demonstrate intrasystem repeatability under controlled conditions with identical stored images, which is fundamentally distinct from real-world clinical reproducibility. In practice, ultrasound acquisition is inherently operator dependent and influenced by variability in probe positioning, lesion visualization, and imaging parameters. Accordingly, the high repeatability observed in this study does not imply consistent performance in real-world clinical settings.

### Clinical Implications

**Figure 2.**
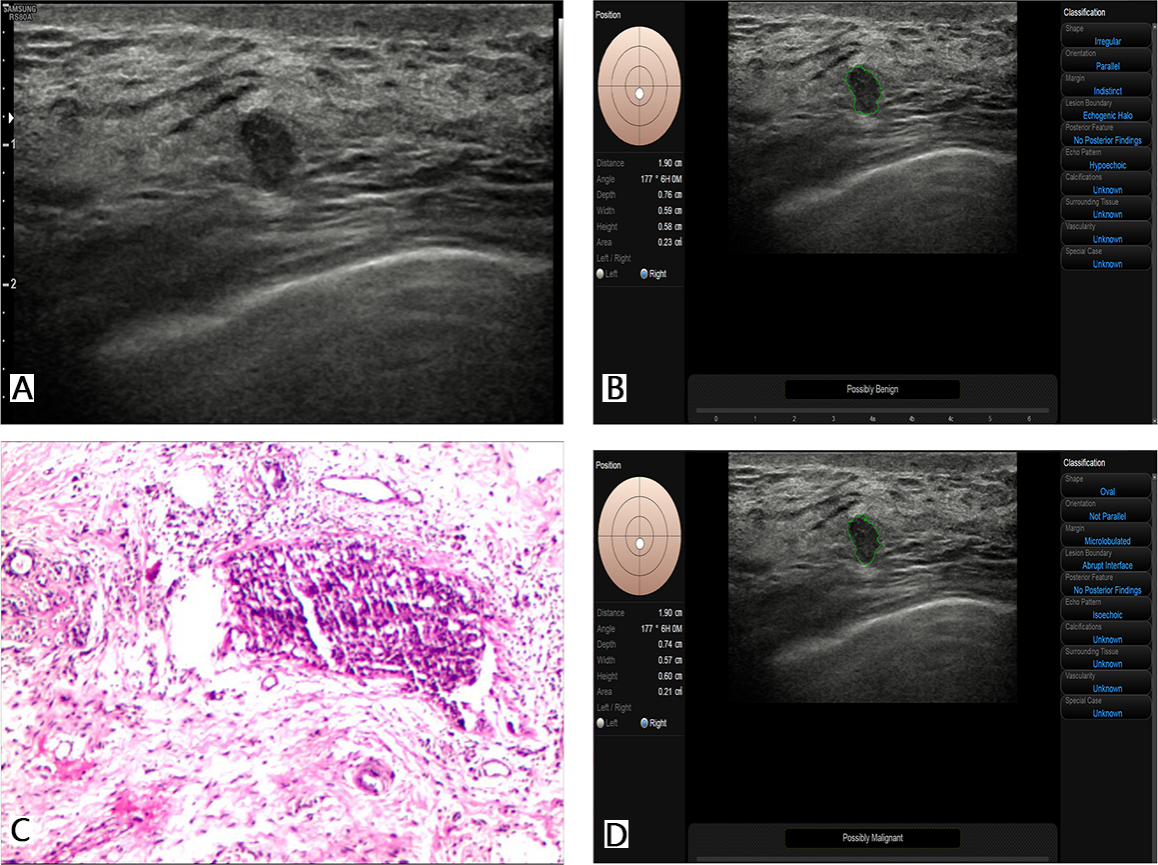
Case 1: a woman aged 27 years was found to have a left breast mass during routine physical examination. Ultrasound revealed a hypoechoic lesion measuring approximately 0.60 × 0.57 cm in the glandular layer beneath the left nipple, adjacent to the areola. The lesion exhibited relatively circumscribed margins and a slightly irregular shape, with a nonparallel orientation. No posterior acoustic attenuation or architectural distortion of the surrounding tissue was observed (A). The initial S-Detect assessment suggested a benign lesion (B), whereas the repeated assessment indicated possible malignancy, demonstrating discordant results (D). Postoperative pathology confirmed usual ductal epithelial hyperplasia with inflammatory cell infiltration (C).

**Figure 3.**
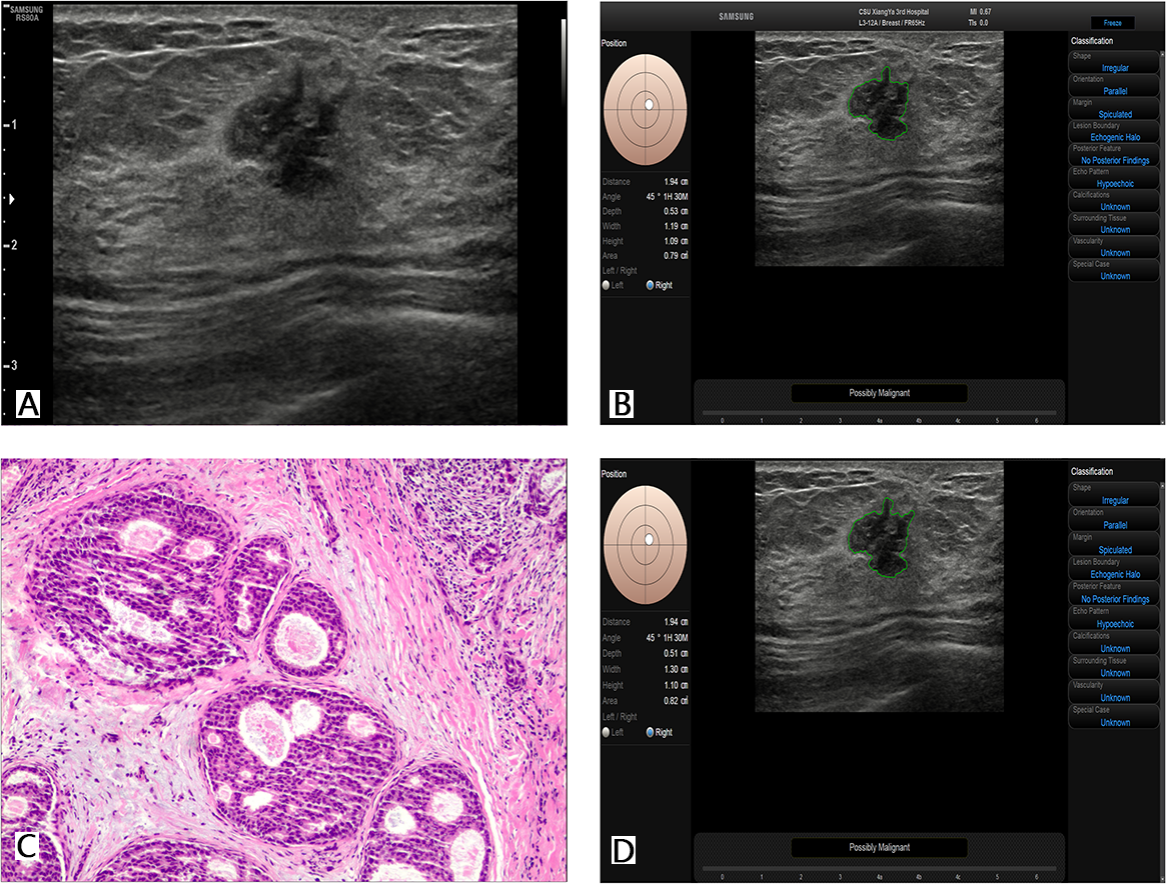
Case 2: a woman aged 43 years presented with a self-detected right breast mass. Ultrasound revealed a hypoechoic lesion measuring approximately 1.30 × 1.09 cm in the glandular layer of the upper inner quadrant of the right breast, adjacent to the areola. The lesion exhibited indistinct margins, an irregular shape, and a nonparallel orientation. No significant posterior acoustic attenuation was observed, whereas architectural distortion of the surrounding tissue was present (A). The initial S-Detect assessment suggested malignancy (B), and the repeated assessment yielded consistent findings (D). Postoperative pathology confirmed invasive ductal carcinoma with focal necrosis (C).

The malignancy prevalence in this cohort was substantially higher than that expected in screening populations. Notably, all discrepant cases in our cohort were upgraded from “possibly benign” to “possibly malignant” upon repeat analysis, and no cases were downgraded from “possibly malignant” to “possibly benign.” This conservative tendency likely reflects algorithmic thresholds designed to minimize false negative classifications, an approach that is clinically preferable for a screening or triage adjunct, although it may increase false positives as shown in case 1 (Figure 2) and case 2 (Figure 3). In practical terms, high intrasystem repeatability supports integration of S-Detect into routine workflows as a consistent second reader, with potential to reduce intraobserver variability; standardize lesion triage; and facilitate training in settings with heterogeneous levels of expertise, such as community hospitals and teaching environments [24-26].

### Strengths and Limitations

A major strength of this study lies in the use of standardized image acquisition protocols and a controlled reanalysis workflow, which minimized operator-related variability and enabled a rigorous assessment of intrasystem repeatability.

Several limitations should nevertheless be acknowledged. First and most importantly, this was a single-center study based exclusively on images acquired using 1 Samsung RS80A Ultrasound system with a single software version, and all analyses were performed on the same workstation. Consequently, the observed repeatability reflects performance under highly homogeneous technical conditions, and the generalizability of these findings to other ultrasound platforms, software versions, or institutional settings remains uncertain. Second, all images were acquired by a single experienced sonographer, and interoperator variability was not evaluated. Accordingly, the repeatability of S-Detect across operators with different levels of experience remains unclear. Third, the study was limited to the analysis of identical stored static images rather than real-time ultrasound examinations. In clinical practice, ultrasound acquisition is inherently operator dependent and influenced by variations in probe positioning, angulation, lesion visualization, and patient respiration, all of which introduce sources of variability absent from this study design. Therefore, these findings should be interpreted as reflecting software-level consistency rather than procedural reproducibility in real-world clinical settings. Finally, the study cohort exhibited a relatively high prevalence of malignancy (228/398, 57.3% of the nodules), substantially exceeding that typically observed in screening populations. This likely reflects referral bias at a tertiary care center and may have inflated PPVs while limiting the applicability of NPVs and diagnostic accuracy estimates to lower-risk screening settings.

### Future Directions

Future studies should adopt multicenter, prospective designs with larger and more diverse cohorts and include multi-vendor comparisons to determine whether observed behaviors are specific to S-Detect or generalizable across CAD implementations. They should evaluate repeatability across different ultrasound platforms and software versions, including longitudinal stability after software updates, and assess CAD performance and stability during real-time scanning across operators with varying levels of experience and at multiple centers. Finally, studies should integrate repeatability and diagnostic accuracy analyses to deliver a comprehensive assessment of CAD performance for clinical deployment.

### Conclusions

The S-Detect CAD system demonstrates high intrasystem repeatability when analyzing identical static ultrasound images under controlled conditions. These findings indicate stable software performance but do not directly translate to real-world clinical reproducibility. Further multicenter and real-time studies are required to validate its performance in routine clinical practice.

## Supplementary material

10.2196/86278 Multimedia Appendix 1 Original ultrasound image.

10.2196/86278 Multimedia Appendix 2 Cursor placement.

10.2196/86278 Multimedia Appendix 3 Automatic region of interest generation.

10.2196/86278 Multimedia Appendix 4 Final result.

10.2196/86278 Multimedia Appendix 5 Artificial intelligence system operation demonstration video.

## Abbreviations

AUC: area under the curve
CAD: computer-aided diagnosis
NPV: negative predictive value
PPV: positive predictive value
