# Supplementary figures and images for: Intrasystem Repeatability of S-Detect for Breast Ultrasound Classification With Identical Static Images: Single-Center Retrospective Repeatability Study
Source: JMIR Med Inform. 2026 Jul 3;14:e86278. doi: 10.2196/86278 (PMC13331062; doi:10.2196/86278)

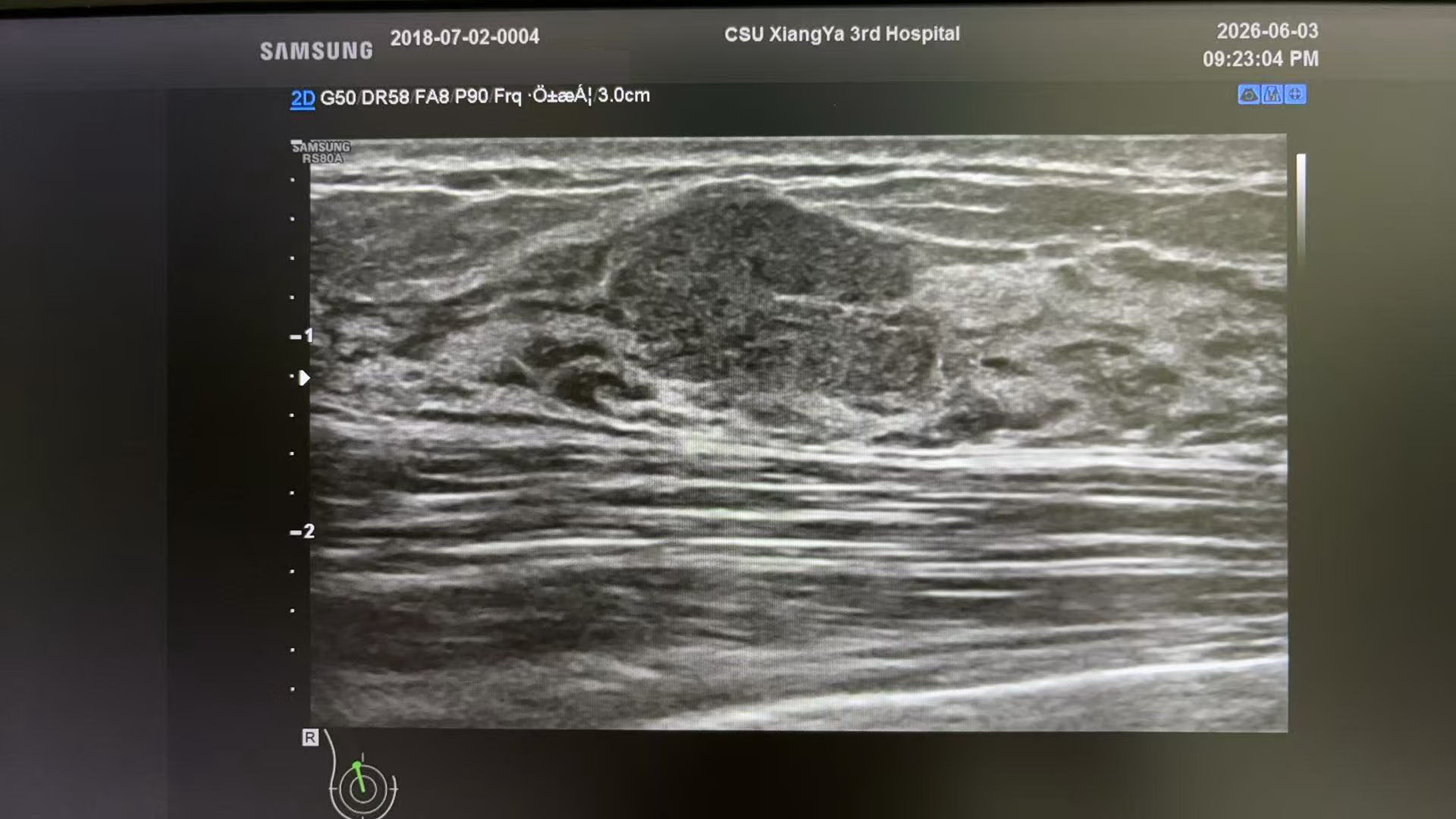

Supplement: Multimedia Appendix 1 [file medinform-v14-e86278-s001.png]

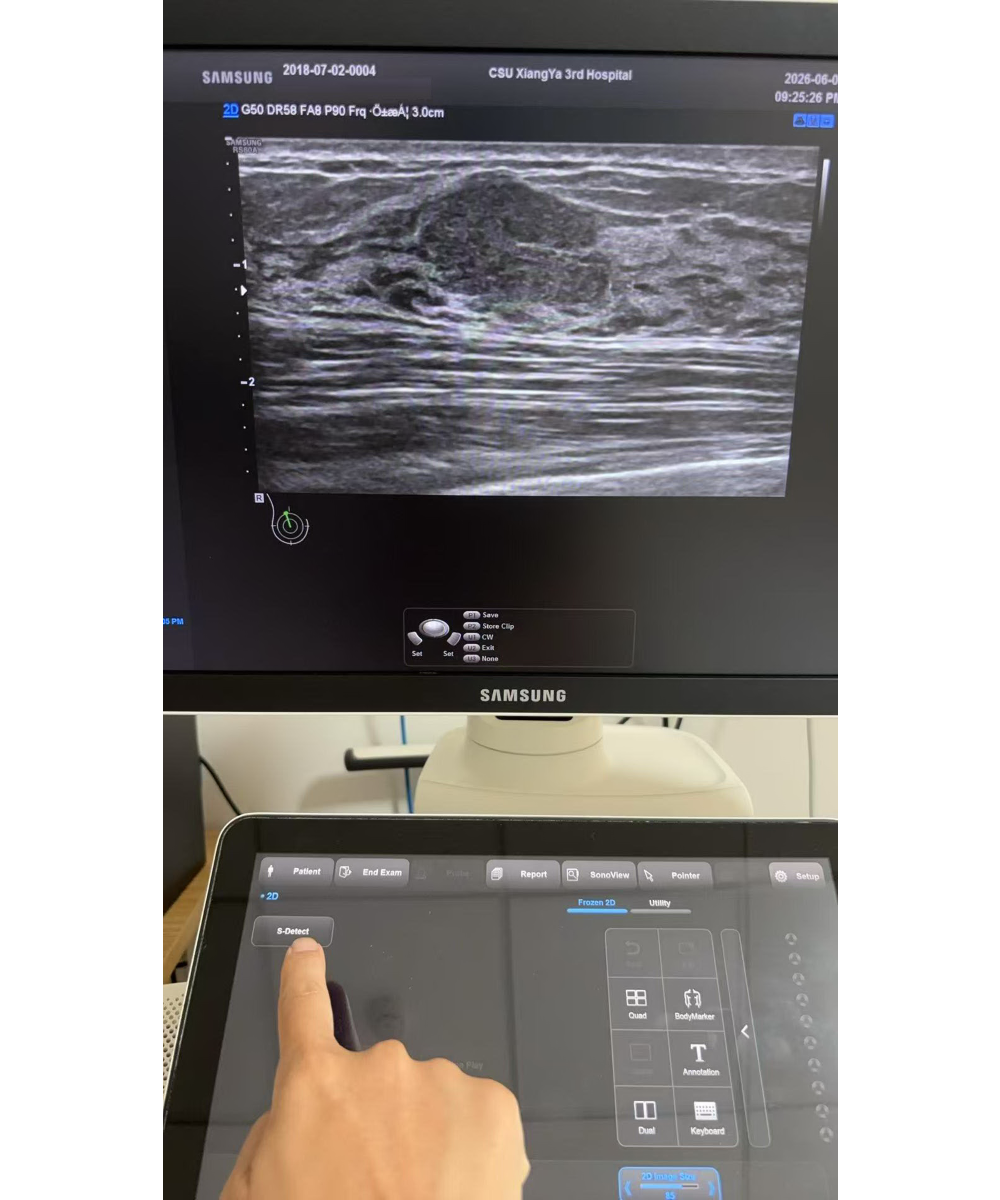

Supplement: Multimedia Appendix 2 [file medinform-v14-e86278-s002.png]

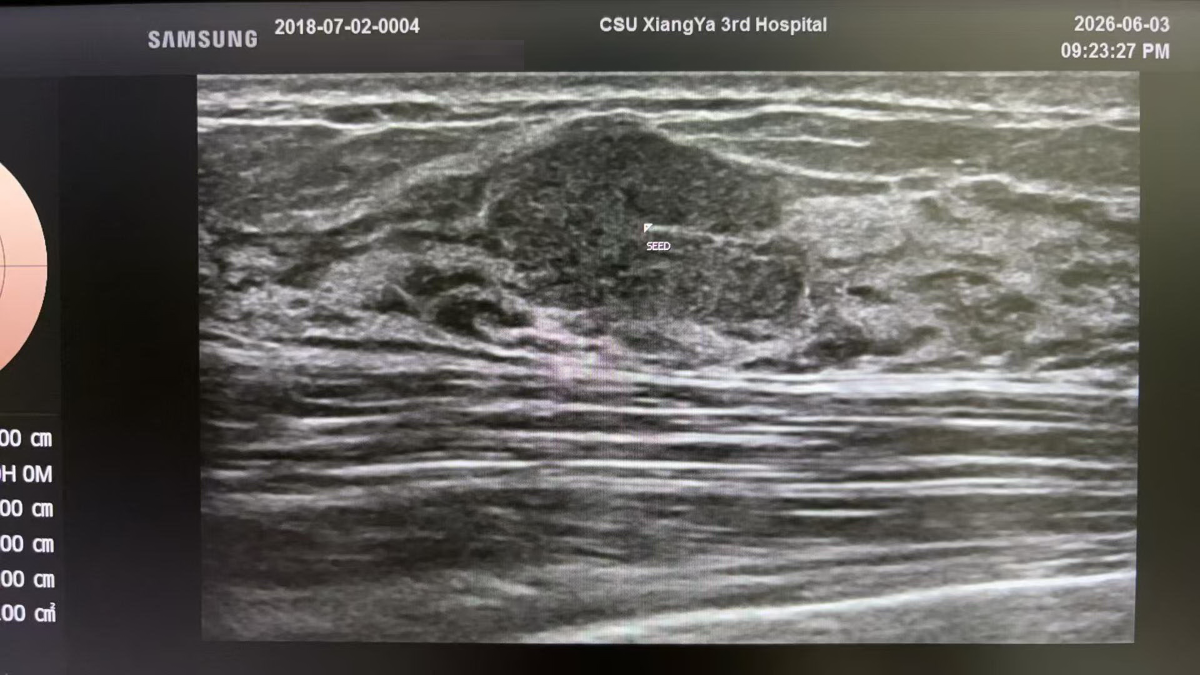

Supplement: Multimedia Appendix 3 [file medinform-v14-e86278-s003.png]

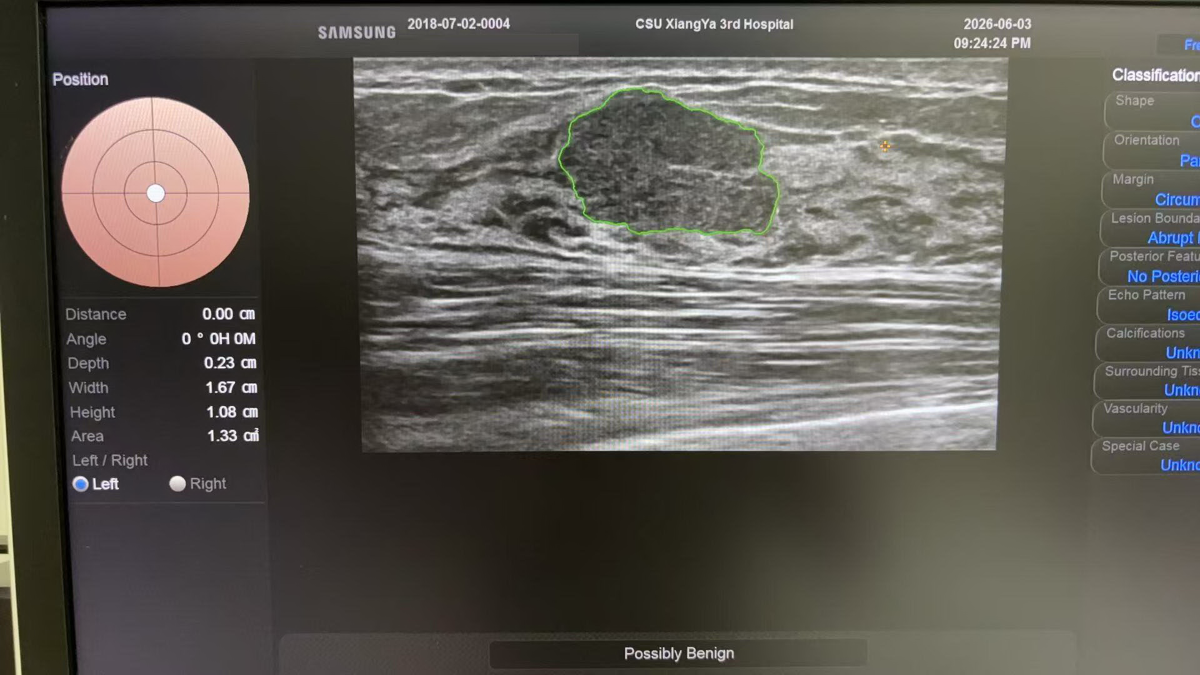

Supplement: Multimedia Appendix 4 [file medinform-v14-e86278-s004.png]
